# Dominant-Negative CK2α Induces Potent Effects on Circadian Rhythmicity 

**DOI:** 10.1371/journal.pgen.0040012

**Published:** 2008-01-18

**Authors:** Elaine M Smith, Jui-Ming Lin, Rose-Anne Meissner, Ravi Allada

**Affiliations:** 1 Department of Neurobiology and Physiology, Northwestern University, Evanston, Illinois, United States of America; 2 Northwestern University Interdepartmental Neuroscience Program, Northwestern University, Evanston, Illinois, United States of America; The Rockefeller University, United States of America

## Abstract

Circadian clocks organize the precise timing of cellular and behavioral events. In *Drosophila*, circadian clocks consist of negative feedback loops in which the clock component PERIOD (PER) represses its own transcription. PER phosphorylation is a critical step in timing the onset and termination of this feedback. The protein kinase CK2 has been linked to circadian timing, but the importance of this contribution is unclear; it is not certain where and when CK2 acts to regulate circadian rhythms. To determine its temporal and spatial functions, a dominant negative mutant of the catalytic alpha subunit, CK2α*^Tik^*, was targeted to circadian neurons. Behaviorally, CK2α*^Tik^* induces severe period lengthening (∼33 h), greater than nearly all known circadian mutant alleles, and abolishes detectable free-running behavioral rhythmicity at high levels of expression. CK2α*^Tik^*, when targeted to a subset of pacemaker neurons, generates period splitting, resulting in flies exhibiting both long and near 24-h periods. These behavioral effects are evident even when CK2α*^Tik^* expression is induced only during adulthood, implicating an acute role for CK2α function in circadian rhythms. CK2α*^Tik^* expression results in reduced PER phosphorylation, delayed nuclear entry, and dampened cycling with elevated trough levels of PER. Heightened trough levels of *per* transcript accompany increased protein levels, suggesting that CK2α*^Tik^* disturbs negative feedback of PER on its own transcription. Taken together, these in vivo data implicate a central role of CK2α function in timing PER negative feedback in adult circadian neurons.

## Author Summary

The molecular mechanism that governs organization of physiology and behavior into 24-h rhythms is a conserved transcriptional feedback process that is strikingly similar across distinct phyla. Notably, cyclic phosphorylation of negative feedback regulators is critical to time molecular rhythms. Indeed, mutation of a putative phosphoacceptor site in the human *PERIOD2* gene, a key negative regulator, is associated with Advanced Sleep Phase Syndrome. This study reveals a critical role for the protein kinase CK2 for setting the period of behavioral and molecular oscillations in *Drosophila*. Circadian phenotypes due to CK2 disruption are due to a direct requirement in adult circadian pacemakers. These findings further demonstrate that CK2 modification of the negative feedback regulator PERIOD alters its cyclical phosphorylation, protein abundance, nuclear translocation, and transcriptional repression activity. These studies place CK2 as a central kinase in circadian timing.

## Introduction

Circadian rhythms that orchestrate daily fluctuations in biochemistry, physiology and behavior are observed across distinct phylogenetic kingdoms. Underscoring the evolutionary importance of these clocks, the molecular processes that drive circadian rhythms are also highly conserved. At the core of the circadian pathway is a transcriptional feedback loop. In Drosophila melanogaster, CLOCK (CLK) and CYCLE (CYC) activate expression of target genes such as *period* (*per*) and *timeless* (*tim*) [1–3]. PER and TIM ultimately translocate to the nucleus and inhibit CLK/CYC transcription [3–6]. Notably, the overall architecture of this feedback loop, as well as some of the molecular players, are observed in organisms as diverse as fungi, plants, and mammals [3].

In addition to transcriptional influence in circadian rhythms, posttranslational modification, particularly for PER, has been shown to play a critical role in normal and disordered circadian timing [7–10]. The most well studied kinase CK1/DOUBLETIME (DBT) is hypothesized to regulate PER nuclear entry, repression, and degradation [7,11,12]. A second enzyme, glycogen synthase kinase (GSK3β)/SHAGGY (SGG), regulates phosphorylation of TIM protein, levels, and nuclear entry [13]. These rhythmic phosphorylation cycles also necessarily include the activity of a phosphatase, and protein phosphatase 2A (PP2A) has been implicated in the rhythmic dephosphorylation of PER [14], while protein phosphatase 1 (PP1) has been implicated in the dephosphorylation of both TIM and PER [15].

Our laboratory has been investigating the function of the protein kinase CK2 in circadian clock function [16,17]. The CK2 holoenzyme is a heterotetramer consisting of two alpha catalytic and two beta regulatory subunits [18,19]. Mutant CK2α and CK2β alleles result in period lengthening phenotypes (<3 h long), consistent with their proposed clock role [16,20]. The manner in which CK2 is important for setting circadian period remains unclear. CK2 also functions in various developmental processes [18], consistent with the pre-adult lethality observed in CK2α and CK2β mutants [16,21]. This developmental function raises the question that CK2 phenotypes may derive from its activity during maturation rather than in adults. While both CK2 subunits are expressed in pacemaker neurons [16,20], it is uncertain if CK2 functions in these neurons to regulate circadian rhythms. RNAi studies in S2 cells suggest that the role of CK2 phosphorylation is to promote transcriptional repression by PER [22]; however, it is not clear if this is true in vivo.

To better address these questions, we expressed a dominant negative CK2α *Timekeeper* (*Tik*) mutant [16] in a spatially and temporally controlled manner and queried the effects on behavior, PER protein levels, phosphorylation, repression, and nuclear entry in core pacemaker neurons of adult D. melanogaster. Taken together, these findings reveal remarkably potent effects of manipulating CK2 activity in adult circadian neurons and uncover a role consistent with the regulation of PER nuclear localization and feedback repression.

## Results

### Induction of the Dominant CK2α*^Tik^* Mutation in Circadian Neurons Dramatically Lengthens Circadian Period

Prior studies implicate CK2 in the control of circadian function in *Drosophila*, *Arabidopsis*, and *Neurospora* [16,20,23,24]. Testing of the strongest homozygous mutants alleles is limited by developmental lethality [16,21]. More modest period phenotypes raised questions as to the functional significance of CK2 action in the circadian clocks. To determine the consequences of suppressing CK2 activity, we used the GAL4/UAS system to drive expression of CK2α bearing the dominant *Tik* mutation (CK2α*^Tik^*) [25]. The CK2α*^Tik^* allele contains two missense mutations, one of which introduces a charged residue into the putative hydrophobic binding pocket for the phosphodonor nucleotide [16,19]. In vitro analysis indicates that these mutations eliminate most catalytic activity [26]. The molecular lesion, the loss of biochemical activity and the dominant behavioral phenotype suggest that *Tik* encodes a dominant negative form of CK2α.

**Figure 1:**
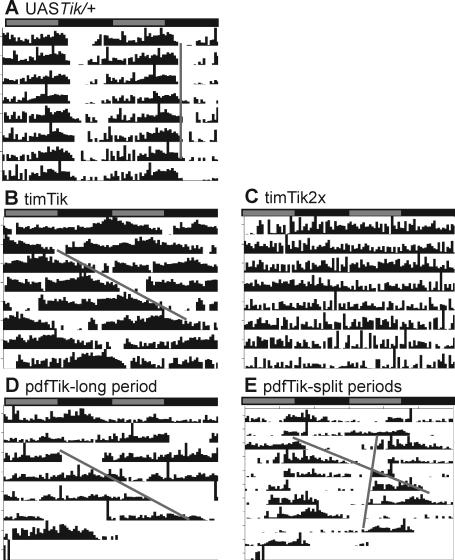
Circadian CK2α Loss of Function Alters Period and Rhythmicity (A–E) Representative double-plotted actograms of indicated genotypes. The *x*-axis symbolizes two consecutive 24-h time frames; gray bars, subjective day; black bars, subjective night. The *y*-axis represents consecutive days and vertical black bars represent activity of a single fly. (A) The UAS*Tik* alone control shows a normal 24-h period. (B) Expression of a single copy of UAS*Tik* with *tim*Gal4, a broad circadian driver, lengthens period while (C) increased transgene dosage induces behavioral arrhythmicity. (D) Overexpression of dominant-negative CK2α in PDF-positive clock neurons causes long periods or (E) period splitting.

To examine the behavioral consequences of CK2α*^Tik^* expression, we crossed flies bearing UAS-driven CK2α*^Tik^* (UAS*Tik*) with *tim*Gal4–62 driver flies [27] and assayed circadian behavior in the progeny (*timGal4/+; UASTik^T1^/+*, “timTik”). The *Drosophila* circadian network consists of six bilateral groups of cells: large and small ventral lateral neurons (lg- and sm- LNv), dorsal lateral neurons (LNd), and three clusters of dorsal neurons (DN1–3) [28]. The *tim* promoter induces GAL4 expression in all of these key neuronal clusters that coordinate circadian behavior [29]. To our surprise, these timTik flies display extraordinarily long periods averaging ∼33 h relative to control periods of ∼24 h (Figure 1, compare Figure 1A and Figure 1B; Table 1). Moreover, the influence on period is dose-dependent; by increasing Gal4 dosage in timTik flies with a second circadian driver, *cry16*Gal4 [30], the period is further lengthened to ∼37 h (Table 1). Confirming the circadian specificity of this result, expression of UAS*Tik* only in photoreceptor neurons with the GMRGal4 driver [31] does not result in period lengthening (data not shown). Heterozygous *Tik/+* mutant flies display periods 2–3 h longer than wild-type controls with a reduction of ∼50% in CK2 activity [16]. The magnitude of the period effects strongly argues that CK2 activity is more gravely inhibited in timTik flies. The fact that the magnitude of period effects exceeds that of nearly all circadian mutant alleles suggests that CK2 activity is critically important for setting circadian period.

**Table 1:**
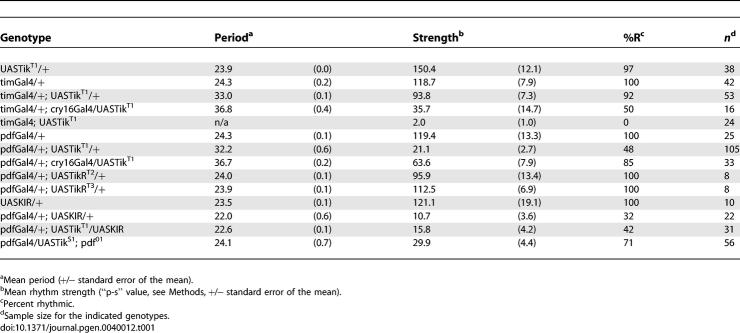
Expression of Dominant-Negative CK2α Alters Circadian Period and Rhythmicity

By increasing dosage of the dominant allele with double copies of both the broad circadian *tim*Gal4 driver and the UAS*Tik* transgene (*timGal4; UASTik^T1^*, “timTik2x”), rhythmicity is undetectable in constant darkness (Figure 1C; Table 1). The above results suggest that CK2α function in central pacemaker neurons is essential for wild type behavioral rhythms. Thus, CK2α and DBT appear to be the only core circadian kinases demonstrated to be obligatory for free-running behavioral rhythms [11,32]. Mutations in a catalytic subunit of the cAMP-dependent protein kinase (PKA) also result in behavioral arrhythmicity [33]; however, as this lesion leaves core molecular cycling of the clock intact, it is likely to function in an output capacity.

### Expression of CK2α*^Tik^* in PDF+ Pacemaker LNv Leads to Robust Period Lengthening and Complex Rhythms

The neuropeptide Pigment-Dispersing Factor (PDF) mediates transmission of timing information from core LNv pacemaker neurons to downstream neural circuits [34]. The CK2α and β subunits are strongly expressed in the pacemaker LNv [16,20]. To test the hypothesis that CK2α functions in pacemaker neurons, CK2α*^Tik^* was induced in the LNv using a *pdf*Gal4 driver [34]. Similar to timTik flies, CK2α*^Tik^* expression in PDF+ neurons (*pdfGal4/+; UASTik^T1^/+*, “pdfTik”) also results in dramatically long periods (∼32 h; Figure 1D; Table 1). Again, these effects are dose-dependent, as adding an additional Gal4 driver, *cry16*Gal4, increased the period length to ∼37 h (Table 1). We previously identified a spontaneous revertant allele, *TikR*, which deletes a portion of the *Tik* coding region, largely reverts the dominant circadian phenotype but still lacks catalytic activity, consistent with its characterization as a recessive loss-of-function allele [16]. Supporting this hypothesis, *pdf*Gal4 expression of independent UAS*TikR* lines had no significant effect on circadian rhythms (Table 1). These results confirm the hypothesis that the *Tik* mutation acts as a dominant-negative to inhibit function of endogenous wild type CK2α.

PDF neurons communicate with and reset the clocks in non-PDF pacemaker neurons to synchronize different clusters in the network [35]. The CK2α*^Tik^* period effects were blocked in a *pdf* null [34] background or by coexpressing an inwardly rectifying potassium channel that hyperpolarizes the LNv (UASKir2.1, [36], Table 1), indicating that CK2α*^Tik^* period effects are transmitted by LNv activity and PDF output. These manipulations alone (*pdf^01^* null mutants or expression of UASKIR with *pdf*Gal4) result in short, weak periods (Figure S1; Table 1) ([34,36]). These data provide functional evidence that CK2α operates in pacemaker LNv to regulate circadian period, consistent with published expression data.

**Figure 2:**
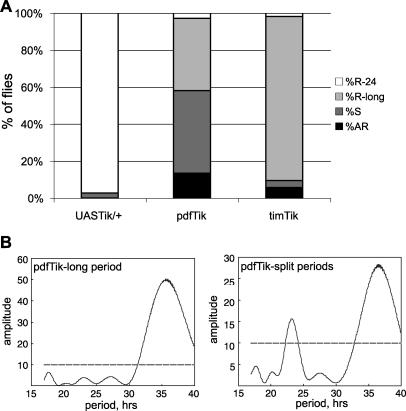
Overexpression of Dominant-Negative CK2α in LNv, but Not All Circadian Neurons, Induces Behavioral Splitting (A) Percentages of flies in each genotype that show a single ∼24-h period (%R-24, white), a single long ∼36-h period (%R-long, light gray), two split peaks of ∼23 h and ∼35 h, (%S, dark gray), and arrhythmicity (%AR, black). UAS*Tik*/+: control, pdfTik: *pdfGal4/+; UASTik^T1^/+*, timTik: *timGal4/+; UASTik^T1^/+*. Overexpression of UAS*Tik* in LNv only causes behavioral splitting in 45% of flies; such splitting is not observed when all circadian neurons are subjected to UAS*Tik* expression. (B) Representative Lomb-Scargle periodograms depicting *pdfGal4/+; UASTik^T1^/+* flies with a single long period (left) or split periods (right).

While pdfTik flies show a long period phenotype, we also noted variability in the period measurement and reduction of the strength of the rhythm in these flies (Table 1). It was hypothesized that “wild-type” non-PDF clock neurons were unable to entrain to the long period program in PDF+ cells, and were expressing a secondary rhythm. To see if flies were exhibiting more than one period, we performed periodogram analysis using the Lomb-Scargle method [37,38]. This approach eliminates misidentification of periods that are simply multiples of a true period. This analysis reveals that approximately 45% of pdfTik animals display two significant periods (Figures 1E and 2A). The dominant period is 35.3 (+/− 0.3) h while a secondary peak indicates an average period of 23.2 (+/− 0.1) h (Figures 2B and S2). When UAS*Tik* is expressed as a heterozygote with the broader expressing *tim*Gal4 driver, reduced rhythm strength and splitting is not detectable (Figure 2A; Table 1), suggesting that hyper-elongating period only in PDF-positive LNv causes uncoupling of clock cell groups. We propose that non-PDF neurons are unable to maintain synchrony with PDF clocks with extreme periods. To our knowledge, this is the first example of complex rhythmicity due to altering period length in a subset of pacemaker neurons.

### CK2α Functions in Adult Pacemaker Neurons to Control Circadian Period

**Figure 3:**
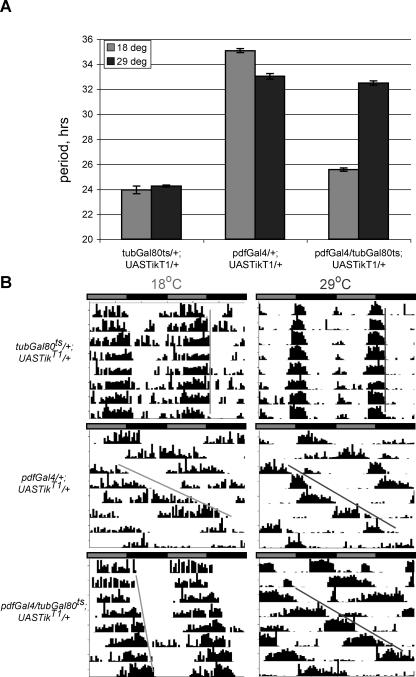
Conditional Induction of CK2α*^Tik^* Induces Period Lengthening during Adulthood Flies were raised at 18 °C to allow Gal80 inhibition of Gal4-mediated transcription and then tested at either the Gal80-permissive (18 °C) or -restrictive (29 °C) temperatures. (A) Average period values. Period lengthening is observed when UAS*Tik* is induced in adult LNv acutely during the testing period. (B) Sample actograms for the indicated genotypes at Gal80-permissive or -restrictive temperatures.

Given that CK2 acts in multiple pathways throughout the life cycle of the fly, we queried if the CK2α*^Tik^* phenotype is due to developmental/compensatory effects or whether loss of CK2α function during adulthood would still result in lengthening of period. In order to address this concern, we utilized a temperature sensitive Gal80 inhibitor of Gal4 expressed in all cells under the tubulin promoter (tubGal80^ts^, [39]). This conditional, temporal and regional gene expression targeting (TARGET) system has been previously used to examine the genetic basis of complex behaviors such as memory in *Drosophila* [39]. tubGal80^ts^ represses GAL4 at the permissive temperature of 18 °C, but is inactivated and fails to repress GAL4 at the restrictive 29 °C temperature. We generated *pdfGal4/tubGal80^ts^; UASTik^T1^/+* flies and raised them at the permissive temperature (18 °C) to prevent expression of UAS*Tik* so that CK2α function would be largely intact during development. Flies were then tested at either permissive (18 °C) or restrictive (29 °C) temperatures and period was calculated during constant conditions. A cardinal feature of circadian clocks is their temperature compensation, i.e., period is roughly invariant over a broad temperature range [40,41]. Consistent with this idea, the control strain here (*tubGal80^ts^/+; UASTik^T1^/+*) shows little period change between 18 °C and 29 °C (Figure 3A and 3B, top panels). Constitutively inhibiting CK2α in pdfTik flies again demonstrates the severe period lengthening effect at both temperatures (Figure 3A and 3B, middle panels); splitting of rhythms in these flies is observed at levels similar to those described above, but only at 29 °C (unpublished data). Interestingly, when dominant-negative UAS*Tik* is selectively activated at 29 °C during testing of adult flies, the extreme long period phenotype (>30 h) is still manifested (Figure 3A and 3B, bottom panels). Slight period lengthening is detectable at 18 °C; however, this effect is much smaller than observed at 29 °C, and is likely due to incomplete Gal80 inhibition of Gal4. Additionally, two split periods are again observed at 29 °C in conditionally inhibited circadian CK2α flies, but not at 18 °C (data not shown). These results indicate that CK2α plays a direct role in adult circadian rhythms, and its loss of function in *Tik* and UAS*Tik* animals is not likely due to some developmental artifact. Consistent with this idea, inspection of LNv structure and PDF labeling in UAS*Tik*-expressing brains reveals no gross abnormalities of circadian pacemaker anatomy (unpublished data). To our knowledge, this is one of the few temporal investigations of clock gene function demonstrating an acute role of a circadian gene during adulthood [42,43].

### Inhibition of CK2α Activity in Pacemaker Neurons Delays PER Rhythms

**Figure 4:**
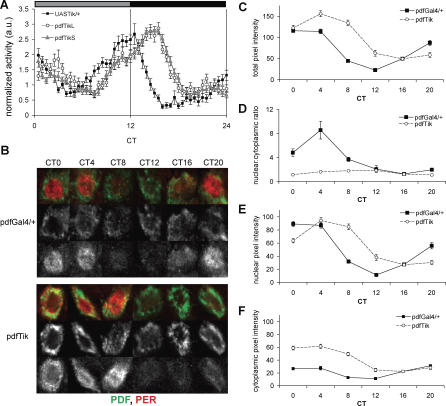
Expression of CK2α*^Tik^* in PDF-positive Pacemaker Neurons Increases PER Levels and Delays Nuclear Accumulation (A) Average behavior activity profile for the first day of constant darkness reveals a delayed evening peak for both long (pdfTikL) and split (pdfTikS) flies expressing UAS*Tik* in PDF+ cells relative to the UAS control alone. (B) Representative images of smLNv cells labeled for PDF (green) and PER (red) in control (*pdf*Gal4/+) and pdfTik (*pdfGal4/+; UASTik^T1^/+*)*.* (C) Quantification of PER levels indicates a delay in the decline of PER protein during middle of the subjective day. (D) Ratio of nuclear:cytoplasmic PER signal in smLNv from control and UAS*Tik-*expressing brains shows a diminished relative nuclear accumulation of PER in pdfTik cells. (E) Average pixel intensity of nuclear PER levels in control and pdfTik smLNv nuclei shows delayed nuclear accumulation with CK2α loss of function. (F) Cytoplasmic pixel intensity demonstrates elevated levels of PER in pdfTik smLNv during early subjective day, suggesting that reduced CK2α activity causes excess PER to be sequestered in the cytoplasm. *n* = 50–76 cells from two independent experiments.

To determine the effects of CK2α loss of function on core molecular clock rhythms, we tested whether expression of UAS*Tik* in PDF-positive LNv altered cycling of the core clock protein PER. Levels and cellular distribution of PER protein in smLNv were examined quantitatively on the first day of DD in pdfTik or control Gal4 flies. Although we do observe splitting in these flies, behavior remains largely synchronous on the first day of DD (Figure 4A). Control flies show the typical evening peak of activity at ∼CT12 while the long-period pdfTik flies have a delayed evening activity peak, regardless of whether they exhibit split periods or not (Figure 4A, pdfTikL v. pdfTikS). Measurements of pixel intensity indirectly report the amount of PER protein in smLNv [44]; as seen in Figure 4B and 4C, PER levels are elevated in smLNv of pdfTik during the subjective day relative to controls. Wild type PER levels wane from CT4–8 and begin accumulating again in the subjective evening (CT16–20); in contrast, a prolonged decline in PER throughout the day (CT4–12) is evident in pdfTik flies, and levels only disappear during subjective evening (CT12–20), consistent with a long period phenotype. Peak and trough PER levels are also elevated in pdfTik flies relative to controls (*p* < 0.001 comparing *pdf*Gal4/+ CT0 to pdfTik CT4 for peak and *pdf*Gal4/+ CT12 to pdfTik CT16 for trough, Figure 4C). PER typically transitions from the cytoplasm to a predominantly nuclear distribution during the middle of the night, and such a pattern is observed in *pdf*Gal4/+ control flies (Figure 4B and 4D). However, the amplitude of the localization rhythm (as quantified by the nuclear:cytoplasmic ratio) is seriously reduced in pdfTik flies (Figure 4D, *p* < 0.001 at CT0, CT4, CT8, and CT20 *pdf*Gal4/+ v. pdfTik). The timing of nuclear localization is also delayed in pdfTik flies; while PER never becomes predominantly nuclear, the time at which the most PER is localized to the nucleus occurs later from CT4–12 in pdfTik smLNv, rather than CT0–4 for the GAL4 control (Figure 4D and 4E). This finding is supported by analysis of nuclear PER levels in *pdf*Gal4/+ and pdfTik smLNv. Nuclear PER levels accumulate to a similar degree in pdfTik as in the Gal4 control; however, nuclear levels do not rise until later in the subjective day relative to control (Figure 4E). The overall fraction of nuclear PER is lower (Figure 4D), as more of the PER protein in pdfTik neurons remains sequestered in the cytoplasm (Figure 4F). Indeed, the reduced nuclear PER levels in the face of elevated cytoplasmic PER levels at CT0 provide the most compelling evidence that CK2α is important for nuclear PER localization independent of regulating its cytoplasmic abundance. These results are consistent with prior reports that reduction of CK2 activity inhibits nuclear entry [16].

### CK2α*^Tik^* Expression Reduces PER Phosphorylation and Increases PER Trough Levels

**Figure 5:**
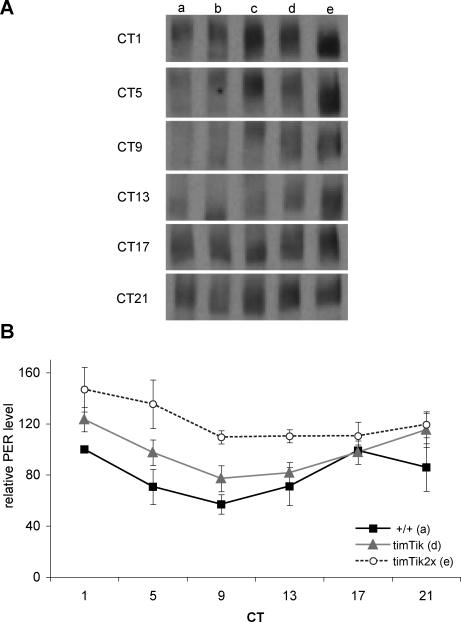
CK2α*^Tik^* Circadian Overexpression Alters PER Protein Levels and Mobility (A) Representative PER western blots demonstrating differences in PER protein amount and mobility with UAS*Tik* overexpression during constant conditions. (a) *y w*, (b) *timGal4/+*; *UASTikR/+*, (c) *Tik/+*, (d) *timGal4/+*; *UASTik^T1^/+*, and (e) *timGal4*; *UASTik^T1^*. (B) Quantification of PER protein levels indicate that decreasing CK2α function results in elevated levels and delayed decline of PER. +/+: *y w*, timTik: *timGal4/+; UASTik^T1^/+*; timTik2x: *timGal4; UASTik^T1^*. CT: circadian time. Quantification done from three independent experiments.

To quantitatively examine the effect of CK2α*^Tik^* on PER cycling and phosphorylation, we used western blots of whole head extracts on the first day of DD. The far majority of PER in whole heads is expressed in the eye [45]. To examine CK2α*^Tik^* effects we used the *tim*GAL4 driver that includes strong expression in the eye. In wild-type flies, PER phosphorylation (evident as reduced mobility) peaks in the early subjective morning (CT1, Figure 5Aa). PER levels are subsequently reduced, presumably reflecting phosphorylation-induced degradation. PER begins to appear early in the subjective night (CT13, Figure 5Aa), and levels accumulate during the night as PER becomes progressively more phosphorylated. In *Tik/+* and timTik flies, both level and mobility rhythms are delayed, consistent with a lengthened period in these flies (Figure 5Ac, 5Ad, and 5B), while expression of UAS*TikR* was not detectably different than wild type (Figure 5Ab).

We then examined flies with two copies of the *tim*GAL4 and UAS*Tik* transgenes (timTik2x). These flies did not exhibit any significant behavioral rhythms (Table 1). Severe reduction of CK2α activity in homozygous timTik2x flies exacerbates PER metabolism during constant conditions, causing constitutive elevations in PER protein and minimizing the amplitude of PER cycling (Figure 5Ae and 5B), consistent with reduced behavioral rhythmicity. These effects are most evident at wild type trough times for PER (*p* < 0.01, significant effect of genotype at CT9). In addition, PER fails to achieve a hyperphosphorylated state in timTik2x flies (Figure 5Ae). This consequence is most evident at CT1, time of peak phosphorylation in wild type. These findings support the notion that CK2α ensures the proper timing of PER cycling and function. While we cannot exclude the possibility that CK2 indirectly regulates the post-translational modification of PER, the strong effects on PER mobility in CK2 loss-of-function flies argue that PER is an in vivo CK2 substrate, consistent with previous studies [17].

### Negative Feedback Repression Is Altered by CK2α*^Tik^* Expression

**Figure 6:**
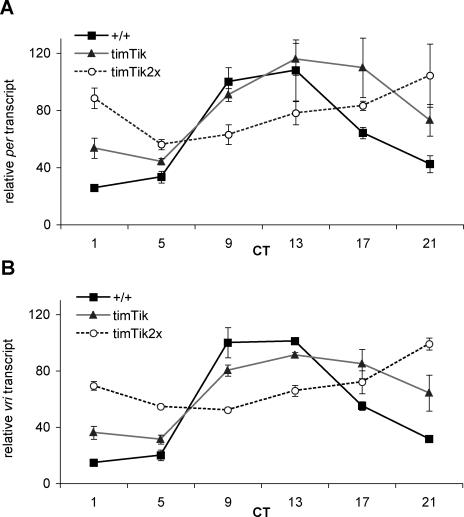
Circadian CK2α Loss of Function Impacts Clock Gene RNA Levels (A) Overexpression of one copy of UAS*Tik* leads to elevated levels of *per* transcript during early subjective night, while further diminishing CK2α function with two copies each of *tim*Gal4 and UAS*Tik* greatly diminishes the amplitude of *per* RNA cycling. (B) Analysis of *vri* transcript reveals a similar pattern of delayed and dampened transcript persistence when CK2α activity is inhibited in circadian cells. +/+: *y w*, timTik: *timGal4/+*; *UASTik^T1^/+*; timTik2x: *timGal4*; *UASTik^T1^*. CT: circadian time. Values obtained from two to three independent experiments.

Previous studies have implicated CK2 in promoting PER repression of CLK activation [22]. However, these studies were performed in cultured *Drosophila* S2 cells which do not harbor functioning circadian clocks. To test the hypothesis that CK2 promotes PER repression in vivo, we examined circadian transcription in UAS*Tik* expressing flies. Levels of two CLK-activated transcripts, *per* and *vrille* (*vri*) [46] were analyzed using quantitative real-time reverse-transcriptase polymerase chain reaction (qRT-PCR). We hypothesized that if negative feedback is unaffected in UAS*Tik* expressing flies, then elevated PER levels would strongly repress CLK, reducing *per* and *vri* transcript levels. If negative feedback is disrupted, then elevated PER levels would fail to appropriately repress *per* and *vri* transcription. Expression of dominant-negative UAS*Tik* in circadian neurons in timTik flies postpones the decline in *per* transcript until early subjective night (Figure 6A), consistent with the effect of CK2α loss of function in the heterozygous *Tik/+* mutant. Whereas wild type *per* transcription peaks around CT9–13, *per* levels do not achieve maximum until CT13–17 in timTik flies, and a similar pattern emerges from analysis of the *vri* transcript (Figure 6B).

The most informative result becomes apparent in timTik2x flies. Further reductions in CK2α activity in timTik2x result in *per* and *vri* transcript levels with a severely reduced amplitude rhythm (Figure 6A and 6B). Importantly, *per* and *vri* never reach wild-type trough levels (*per*: *p* < 0.001 for *y w* at CT1 relative to timTik and timTik2x at CT5 and *p* < 0.01 for *vri* at the same time points), consistent with the hypothesis that elevated PER protein levels are unable to fully repress CLK target genes in UAS*Tik*-expressing flies. Taken together, the magnitude of the observed effects suggests that CK2 not only promotes PER repression activity in vivo, but that it has a sizable impact on transcriptional repression.

## Discussion

The role of posttranslational modification in regulating precise circadian timing is well established [9], and indeed may be principally responsible for molecular cycling [8]. CK2 has been implicated in regulating circadian rhythms, PER modification and metabolism [16,17]. The present study sought to determine if CK2α activity is required in adult core pacemaker neurons for molecular and behavioral rhythmicity. Broad spatial expression of UAS*Tik* in pacemaker neurons with the *tim*Gal4 driver causes severe lengthening of circadian period to ∼33 h, a degree even greater than that of the heterozygous *Tik* mutant. Radical reductions in CK2α activity by increasing copy number of the transgenes in timTik2x flies ultimately result in behavioral arrhythmicity, demonstrating that CK2α is an obligatory component of circadian rhythms. Previous work demonstrated that overexpression of wild type CK2 mildly lengthens period [17]; taken together, these data indicate that period is highly sensitive to CK2 activity. Expression of UAS*Tik* in PDF+ LNv is also sufficient to lengthen period; indeed, the effect requires LNv activity and output of the PDF neuropeptide. That the period length is not exacerbated by additional clock neuron expression in timTik versus pdfTik flies implies that the phenotype originates largely from the LNv; however, the possibility that CK2α additionally functions in other circadian cells cannot be excluded. Given that splitting is eliminated when the genetic programs of both LNv and downstream circadian neurons are identical with respect to UASTik expression, the data imply that this manipulation affects CK2 function in other clock cells. Indeed, if CK2 is a true component of the core transcriptional pacemaker, it would be expected to regulate feedback in all cells that have a functional molecular clock. As CK2 functions to dictate period in LNv cells during constant conditions, it is possible that CK2 activity may also regulate morning behavior, as these cells drive morning activity, while downstream neurons dictate evening activity [47,48]. However, as CK2 may also function in cells responsible for evening behavior, some balance of CK2 between LNv and non-LNv neurons could favor a strong morning or evening activity phase. The contribution of CK2 activity to morning and evening behavior is currently under investigation.

Further evidence that CK2α activity is important in LNv derives from the finding that inhibition by CK2α*^Tik^* causes delayed nuclear entry of PER in these core pacemaker cells. An unanticipated consequence of pdfTik expression is splitting of the behavioral rhythm into long (∼35 h) and short (∼23 h) components. All of the above behavioral effects are due to acute CK2α activity as adult-specific inhibition is able to induce the rhythm phenotypes. At the molecular level, elevated levels and diminished phosphorylation of PER protein is associated with reduced CK2α function; this effect on PER protein is further correlated with elevated and delayed transcription of *per* and *vri* clock genes.

The severity of the observed behavioral phenotype places CK2 as a critical regulator of circadian rhythms. Of known circadian kinases, only mutants of *doubletime* (*dbt*) and PKA are also capable of completely eliminating rhythmicity as is observed in timTik2x flies [32,33]. As the core molecular clock is unperturbed by PKA mutations, this kinase is proposed to function in circadian locomotor output [32,33], leaving DBT and CK2 as the only critical core circadian kinases. Originally, DBT was found to regulate PER stability and electrophoretic mobility; this initial study concluded that DBT-mediated phosphorylation led to PER degradation [11,49]. Subsequent studies suggested that DBT may retard the ability of PER to enter the nucleus and repress transcription [12,22,50]. Many other gene mutations that result in arrhythmicity affect either input or output of the circadian system. As the core molecular feedback loop is disrupted in UAS*Tik*-expressing flies, CK2 appears to regulate timing of the core clock and shows phenotypes similar to mutants of other core circadian genes such as *per*, *tim*, and *Clk*. However, the magnitude of the period phenotype in both pdfTik and timTik flies is greater than nearly all circadian mutants. The only other alleles which produce a similar degree of period lengthening include the *tim^UL^* mutation [51] and a novel dominant-negative kinase dead *dbt* allele whose expression also results in period lengthening or arrythmicity [52].

We present numerous pieces of evidence to support the hypothesis that CK2α acutely functions in the PDF+ LNv neurons. The long period phenotype observed when CK2α*^Tik^* is expressed in PDF-positive LNv is associated with splitting of the behavioral rhythm into two components: a predominant, long, ∼35 h period and a weak shorter period of approximately 23 h. The splitting is reflected in the low strength of behavioral rhythms observed in pdfTik flies. Splitting was originally observed in Syrian hamsters maintained under constant light; this finding was the foundation for a two-oscillator model whose coordinated output is manifested as an overt circadian rhythm [53]. It has similarly been shown that non-conventional entrainment conditions can induce multi-period splitting and desynchronization of circadian neurons in mammals [54,55]. Early reports indicated splitting of the *Drosophila* circadian period in *sine oculis* mutants that have disrupted optic development, suggesting that dual periods may arise from entrainment through different input pathways [56]. Similarly, both wild type flies under low light and mutants of *cryptochrome*, the major circadian photoreceptor, exhibit split rhythms under constant light [57,58]. These periods include short (∼22 h) and long (∼25 h) components that alternatively decrease or increase with light intensity, respectively, again implicating variation of the oscillator system input pathway. Ectopic misexpression of the PDF output neuropeptide induced multiple periods during DD (of ∼22 h and ∼25 h) [59]. Nitabach et al. [38] further identified complex rhythmicity by activating LNv neurons; at least two periods of ∼22 or ∼25–26 h lengths are observed (with an occasional 3^rd^, shorter ∼20–21 h peak). Elevated PDF levels and desynchronization of circadian neurons are detected in these flies [38]. Both of the above cases suggest that split periods arise from misregulation of neuronal output from the core pacemaker neurons. The result presented here is the first demonstration of splitting as a consequence of altering a core clock component.

It is hypothesized that driving the oscillator period to such an extreme only in the LNv uncouples them from non-UAS*Tik* expressing, PDF-negative “wild type” circadian neurons (i.e., LNd/DNs) that then contribute the shorter, weaker behavioral rhythm (∼23 h), such as that seen in *pdf^01^* mutants [34]. This notion is further supported by the behavior of timTik flies that express UAS*Tik* in all circadian neurons; in this case, when the genetic programs of all clock cells are identical, no such splitting is observed and rhythm strength returns to normal levels. These results begin to examine the limits of entrainment of one oscillator by a coupled oscillator in a circadian pacemaker network.

CK2 has a number of roles in cellular biology [18]. It is required at multiple transitions during the cell cycle including mitosis and functions to regulate caspase-mediated apoptosis and cell survival [18]. Developmentally, CK2 regulates proliferation and cell fate decisions [25,60]. Not surprisingly, it is an essential gene, as homozygous *Tik* mutants are not viable as adults [16]. We were able to utilize the TARGET system [39] to conditionally induce dominant-negative CK2α in adult flies. Interestingly, when CK2α activity was inhibited in LNv solely during adulthood, the behavioral phenotypes are still manifested. Thus, an acute CK2α loss of function impacts rhythmicity in the adult circadian system, presenting it as a critical and direct regulator of the circadian clock. While it has been shown that such adult-specific rescue of *per* is able to restore rhythmicity in *Drosophila* [43], it will be important to investigate the life-stage properties of other circadian genes; for example, *Clk* is also known to have developmental roles [61].

Thus, CK2α is an acute, direct, and essential component of circadian rhythms; we propose that CK2 regulates the core oscillator by phosphorylating PER to promote nuclear entry and repression. There is abundant evidence for PER as a *bona fide* CK2α substrate. CK2α can phosphorylate PER in vitro at specific predicted CK2α sites [16,17]; moreover, mutation of these CK2α target residues causes period phenotypes similar to the *Tik* mutation when expressed in vivo, demonstrating the functional relevance of this modification on PER activity [17]. Finally, we present here the clear defects in PER mobility observed with a deficiency in CK2α activity. Expressing UAS*Tik* singly or in double dosage with the *tim*Gal4 driver results in increased, hypophosphorylated PER. Indeed, the amplitude of PER cycling appears completely diminished when CK2α is severely inhibited in timTik2x flies. Again, the molecular PER phenotype mirrors the behavioral effect of these manipulations; timTik2x flies are arrhythmic under constant conditions, supporting the idea that CK2α activity is critical for the maintenance of a molecular and behavioral clock.

A further consequence of CK2α loss of function in core pacemaker neurons is a pattern of delayed PER decline, consistent with the long period phenotype observed in these flies. Despite increases in overall and cytoplasmic PER levels, nuclear PER levels are lower relative to wild type during the early subjective day, providing further evidence that nuclear translocation is not strictly driven by protein accumulation [50,62]. The dampened and delayed nuclear entry of PER protein of CK2α*^Tik^*-expressing smLNv provides support that CK2α normally functions to promote PER nuclear translocation. A second possibility is that the high levels of PER protein saturate the nuclear entry pathway, preventing the majority of PER from localizing to the nucleus in pdfTik flies. Yet, the delay in nuclear accumulation is consistent with the hypothesis that CK2α activity typically functions to permit timely PER nuclear entry.

Previous evidence indicated that knock-down of CK2 levels in cultured *Drosophila* S2 cells limits the ability of PER to repress a *Clk*-driven luciferase reporter [22]. It is critical to validate such studies in vivo to determine the true function of the kinase in the circadian system. While one may expect that the increased levels of PER associated with CK2α*^Tik^* expression (particularly at trough time points) would lead to enhanced clock gene repression, we do not see such an effect. Conversely, CK2α inhibition results in delayed *per* and *vri* transcription, and elevated trough transcript levels, confirming that CK2α normally operates to promote repression of clock gene transcription. The features of CK2α function are both in opposition with and complementary to those put forth for the DBT kinase. DBT is thought to retard PER nuclear entry [12] and signal its degradation [11]; in contrast, CK2α appears to promote nuclear entry of PER (and hence repression), but may also influence its turnover.

We have outlined a model of the way in which CK2 promotes repression of circadian transcription developed from existing and currently presented data. We speculate that effects of CK2 on PER nuclear localization may operate through the proposed interval timer described in S2 cells. Based on the interval timer model, PER and TIM heterodimerize in the cytoplasm in a time-insensitive manner [63]; after some lag or upon some signal, they dissociate and enter the nucleus independently [44,63] where PER mediates transcriptional repression. The role of nuclear TIM is yet unclear. Recent work indicates that repression is not achieved merely by physical association of PER with CLK, but perhaps by PER acting as a scaffold to bridge CLK and DBT [64]. It is hypothesized that phosphorylation of CLK by DBT diminishes its transactivating capabilities [64], similar to the model proposed in *Neurospora* [23]. Nawathean et al. conclude that the ability of PER to repress transcription is primarily a function of its nuclear localization, which is, in turn, dependent on phosphorylation [65]; however, they also acknowledge that phosphorylation may secondarily modulate the intrinsic ability of PER to enact repression. While PER is predominantly cytoplasmic in S2 cells (e.g., [65]), altering its subcellular localization by adding a nuclear localization signal or blocking nuclear export increases transcriptional repression; however, this activity is reduced in mutants that lack a critical DBT binding site shown to be important for PER phosphorylation [64,65]. Thus, the data from S2 cells does not resolve whether activity primarily regulates PER nuclear entry or repression, but provides evidence for both functions. The conflicting S2 cell results, particularly for DBT analysis, support the use of our in vivo approach to determine the role of kinase modification on molecular cycling.

We propose that the effects of CK2 loss of function on feedback repression are due, in part, to the inability of PER to properly translocate to the nucleus. Phosphorylation of the PER:TIM heterodimer in the cytoplasm by CK2 may act as the interval-timer signal [63] to dissociate this complex and/or facilitate nuclear entry. Indeed, with reduced CK2α function, PER nuclear accumulation is delayed, and correlates with delayed repression of circadian transcription. DBT functions to induce degradation of free cytoplasmic PER, as well as hyperphosphorylated nuclear PER. We propose that increased and lingering levels of PER in UAS*Tik*-expressing brains is due to an inability of CK2 to signal dissociation of the PER:TIM heterodimer. Persistence of PER in this complex and its failure to independently enter the nucleus protects it from DBT-mediated phosphorylation. The lack of DBT phosphorylation would reduce PER degradation and repressor activity, leading to increased cytoplasmic PER and altered feedback repression. Intrinsic PER repressor function does not appear to be greatly compromised by CK2 loss of function, as liberation of PER after light-induced TIM degradation in timTik2x flies results in robust suppression of *per* RNA (R. Meissner, J. Lin, unpublished observations). As nuclear entry is a critical step in feedback repression, CK2 function is important for the maintenance of a functional molecular and behavioral clock. Alternatively, CK2 may promote PER-TIM dimerization and subsequent nuclear entry. CK2 may facilitate PER's interaction with CLK and thus enhance repression. Lastly, CK2 could function to augment DBT phosphorylation of PER either by modulating DBT activity or by providing a phosphorylated substrate for recognition by DBT, thus controlling PER abundance.

Conditional spatio-temporal expression of this dominant-negative CK2α mutation is a useful tool for in vivo exploration of the myriad roles of CK2 in all aspects of biology. The UAS*Tik* transgene has already been used to dissect features of CK2 function during *Drosophila* eye development [25]. Here, the use of such a strong CK2α allele permits dissection of the molecular, genetic, and neuroanatomical clock. Ultimately, these studies provide a model in which CK2α activity in the core adult pacemaker is critical for the proper timing of the transcriptional feedback loop. The critical role of CK2α in pacemaker function highlights the importance of a concerted post-translational modification scheme to regulate cycling of core clock components in order to manifest precise circadian rhythms at the molecular and behavioral level. Indeed, mutation of a phosphorylation site in the human *per2* gene is responsible for Familial Advanced Sleep Phase Syndrome [10]. As CK2 is highly conserved across kingdoms and has been shown to function in the circadian pathway of other species [23,24], it is a priority to investigate its role in mammalian circadian rhythms.

## Materials and Methods

### Fly stocks.

For generation of UASTik lines, we cut the *Bgl*II and *Xho*I fragment containing *Tik* from pET-*Tik* [16], and cloned it into pUAST to create transgenic flies. All transgenic DNA constructs were sequenced (Applied Biosystems, Foster City, CA) and injected in *y w* embryos at CBRC Transgenic *Drosophila* Core (Charlestown, MA). Transformant lines with inserts on the second or third chromosome were balanced with *y w*;*Bl/Cy0* or *y w*;;*TM2/TM6B*, respectively. Distinct UAS*Tik* insertions are denoted with *S1* or *T1* superscripts for second or third chromosome inserts, respectively. The revertant *TikR* allele was similarly cloned, and UAS*TikR* inserts are also designated with the superscripts *T2* or *T3* indicating two discrete third chromosome insertions.

For additional fly lines, the *pdf*Gal4 and *tim*Gal4–62 drivers [27,34] as well as *pdf^01^* mutants [34] were obtained from Michael Rosbash, Brandeis University. The UAS-Kir2.1 flies are previously described [36] and were acquired from Grae Davis, UCSF. tubGal80^ts^ flies [39] were ordered from the Bloomington Stock Center, Indiana University. Flies were maintained in standard cornmeal-molasses-agar food at 25 °C unless otherwise noted.

### Behavior.

As described [66], male flies aged 2–7 days old were entrained to 2–5 days of 12 h light:12 h dark (LD) and exposed to 7–10 days of constant darkness (DD). Activity patterns were monitored by the *Drosophila* Activity Monitoring system (TriKinetics) in 30 min bins. Clocklab software (Actimetrics) was used to calculate period estimates using a chi-square periodogram (α = 0.01). Rhythm strength was measured as the power of each record minus the significance (“p-s”). Flies were considered rhythmic if they exhibit a p-s value of >10. Visual inspection of actograms was performed to confirm rhythmicity (or lack thereof) in weakly rhythmic flies. Lomb-Scargle periodograms were constructed using Clocklab to score period splitting as previously described [38] except significance was set at α = 0.01. Period peaks were considered if they crossed the significance line; the percent of flies exhibiting a single peak of ∼24 h versus a single long period or two peaks was calculated.

For developmental analysis (tubGal80^ts^ crosses), flies were mated and progeny raised at 18 °C. Flies were then placed into the behavior apparatus and exposed to 5 days of LD and 7–10 days of DD at either 18 °C or 29 °C such that the LD phase allowed Gal4 induction (at 29 °C) while circadian behavior was calculated during the DD phase.

### Immunostaining.

Adult male flies of the indicated genotypes aged 2–10 days were entrained to at least 3 days of LD and shifted to DD. Brains were dissected on the first day of DD as follows (modified from [44]): following brief anaesthetization, flies were pinned to a Sylgard dish and the proboscis was removed. Cold PBS was placed around the wound until all flies for that time point were processed. PBS was replaced with 4% formaldehyde/PBS to fix for 15 min. The brain was then dissected away from the head capsule under cold PBS, fixed again for 20 min, and stored in PBS at 4 °C overnight. Brains were washed 2 × 5 min in PBSTx (PBS+0.3% Triton-X100), blocked 30 min in PBSTG (PBSTx+10% normal goat sera), and incubated in the following primary antibodies overnight: rabbit anti-PER 1:4,000 and rat anti-PDF 1:1,000 [61,67], gifts from M. Rosbash, Brandeis University. Antibody was removed and brains were washed 4 × 10 min in PBSTx, then incubated with secondary goat anti-rabbit-Alexa488 (Molecular Probes, Invitrogen) and donkey anti-rat- Cy3 (Jackson ImmunoLabs) both at 1:500 dilutions for at least 2 h at room temperature. Final washes of 4 × 10 min in PBSTx were performed, brains were rinsed in PBS, and placed in 80% glycerol overnight at 4 °C before mounting.

Immunofluorescence was imaged with a Nikon C1 confocal microscope and analyzed as reported [44]. Briefly, PDF staining was used to determine cytoplasmic/nuclear compartments, and the average pixel intensity for PER staining was measured with Image J (National Institutes of Health). Background levels in nearby pixels were subtracted from the raw data, and total amounts and the nuclear:cytoplasmic PER signal ratio were calculated for each fly using Excel. A t-test was used to determine the significance between total peak and trough PER values for Gal4 controls versus pdfTik at the indicated CT. T-tests were also employed to probe differences in the nuclear:cytoplasmic ratio amongst the genotypes.

### Western blotting.

Quantification of PER western blotting was performed as described previously with NIH Image J [16]. Equal loading and transfer were confirmed with Ponceau S staining of membranes. The dilutions for the primary and secondary antibodies were 1:10,000 and 1:2,000 in TBST buffer (ECL protocol; Amersham Biosciences). Five TBST buffer washes lasting 5 min each were performed following the primary and the secondary antibody incubations. Blocking was achieved with TBST+5% milk (Bio-Rad). The incubation times for blocking, primary and the secondary antibody incubations were as follows: 1 h at room temperature, overnight at 4 °C, and 1 h at room temperature, respectively. ECL reagents were used for immunoassay signals. A single-factor ANOVA compared the effect of genotype at trough time points.

### Quantitative real-time PCR.

Total RNA was isolated from frozen whole heads using TRIzol reagent (Invitrogen) according to the manufacturer's protocol. DNA was removed from RNA extracts using RQ1 DNase from Promega. Real-time PCR reactions were run using the Applied Biosystems 7900HT fast real-time PCR instrument. Data were collected using SDS software version 2.2.1. Data were analyzed using the 2^−ΔCt^ method [68] using *RP49* expression values to normalize for differences in RNA amount among samples. Statistical significance was evaluated for trough transcript levels by comparing the effect of genotype at the indicated CT using a single-factor ANOVA.

For PCR reactions, ∼100 ng RNA were used per reaction. Reactions were prepared using the reagents from the Qiagen QuantiTect SYBR Green RT-PCR kit. Total reaction volume was 25 ml and reactions were run in 96-well plates. Primer sets used were ordered from Integrated DNA Technologies. Primer sequences are as follows. *per*: forward primer is 5′-CAGCAGCAGCCTAATCG-3′, and the reverse primer is 5′-GAGTCGGACACCTTGG-3′. *vri*: forward primer is 5′-TGTTTTTTGCCGCTTCGGTCA-3′, and the reverse primer is 5′-TTACGACACCAAACGATCGA-3′. *RP49*: forward primer is 5′-CGACGCTTCAAGGGACAGTATC-3′, and the reverse primer is 5′-TCCGACCAGGTTACAAGAACTCTC-3′. RTPCR cycling parameters were as follows: 30 min at 50 °C, 15 min at 95 °C, and 30 cycles of 15 sec at 94 °C, 30 sec at 55 °C, and 30 sec at 72 °C.

## Supporting Information

Figure S1 Short, Weak Rhythms Persist in LNv Silenced and *pdf* Null Flies Regardless of UAS*Tik* Expression Sample actograms and P-S rhythmicity values are presented for flies of the following genotypes: pdfGal4/+; UASKIR/+ (pdfKIR), pdfGal4/+; UAS*Tik^T1^*/UASKIR (pdfTikKIR), and pdfGal4/UAS*Tik^S1^*; *pdf^01^* (pdfTik*pdf^01^*).
(635 KB EPS)

Figure S2 Evidence of Split Rhythms in pdfTik Flies (A) Representative actograms (left) and Lomb-Scargle periodograms (right) for pdfTik flies exhibiting short and long periods.
(B) Averaged actogram (left) and Lomb-Scargle periodogram (right) for all split pdfTik flies (*n* = 46).
(1.3 MB EPS)

### Accession Numbers

Following are the accession numbers and FlyBase identifiers for the genes and protein products in this study. National Center for Biotechnology Information (NCBI) Entrez (http://www.ncbi.nlm.nih.gov/Entrez/) gene numbers: *CK2alpha*, GeneID 48448; *period*, GeneID 31251; *vrille*, GeneID 33759. FlyBase (http://flybase.org/) identifiers: *CK2alpha*, FBgn0000258; *CK2alpha^Timekeeper^*, FBal0141857; *CK2alpha^TimekeeperRevertant^*, FBal0141856; *period*, FBgn0003068; *vrille*, FBgn0016076. NCBI Entrez protein database numbers: *Drosophila* CK2alpha (isoform D), NP_001036624; *Drosophila* PERIOD, NP_525056.
